# Genome Sequences of *Pseudomonas* spp. Isolated from Cereal Crops

**DOI:** 10.1128/genomeA.00209-13

**Published:** 2013-05-09

**Authors:** Donald M. Gardiner, Jiri Stiller, Lorenzo Covarelli, Magdalen Lindeberg, Roger G. Shivas, John M. Manners

**Affiliations:** CSIRO Plant Industry, Queensland Bioscience Precinct, St. Lucia, Brisbane, Queensland, Australiaa;; Department of Agricultural and Environmental Sciences, University of Perugia, Perugia, Italyb;; Department of Plant Pathology and Plant Microbe Biology, Cornell University, Ithaca, New York, USAc;; Queensland Department of Agriculture, Fisheries, and Forestry, Ecosciences Precinct, Dutton Park, Queensland, Australiad;; CSIRO Plant Industry, Black Mountain, Canberra, Australian Capital Territory, Australiae

## Abstract

Compared to those of dicot-infecting bacteria, the available genome sequences of bacteria that infect wheat and barley are limited. Herein, we report the draft genome sequences of four pseudomonads originally isolated from these cereals. These genome sequences provide a useful resource for comparative analyses within the genus and for cross-kingdom analyses of plant pathogenesis.

## GENOME ANNOUNCEMENT

Recent comparative genomics analyses of fungi have revealed the importance of cross-kingdom lateral gene transfers in the evolution of the virulence of eukaryotic microbes (1–3). For example, we have identified virulence-associated genes specific to cereal fungal pathogens where phylogenetic analysis strongly indicates that they have been acquired by horizontal gene transfer from bacteria (2). Moreover, in some cases, the most similar sequences available in public repositories to the fungal genes are from plant-associated bacteria. However, the availability of genome sequences of cereal-associated bacteria, particularly those associated with wheat and barley, is limited compared to the availability of genome sequences of dicot-associated bacteria. Herein, we report the genome sequences of four *Pseudomonas* spp. isolated from cereals in Australia to augment the availability of cereal-associated bacterial genomes for comparative analyses.

Cultures of *Pseudomonas* associated with wheat or barley were selected from the Queensland Plant Pathology Herbarium (BRIP). The isolates *Pseudomonas syringae* BRIP 34876 and BRIP 34881 and *Pseudomonas fluorescens* BRIP 34879 were originally isolated from barley glumes showing rot symptoms in September 1971 near Warwick, Queensland, Australia. *P. syringae* isolate BRIP 39023 was isolated from wheat glumes in September 1988 near Bauhinia Downs, Queensland, Australia. In reinoculation experiments on barley and wheat leaves, the *P. syringae* isolates BRIP 34876 and BRIP 34881 resulted in symptom development in both hosts, while *P. fluorescens* BRIP 34879 and *P. syringae* BRIP 39023 did not induce symptoms.

DNA for genome sequencing was prepared from bacteria grown in liquid culture using a Qiagen blood and tissue kit, according to the manufacturer’s instructions. Illumina library preparation and sequencing were performed by the Australian Genome Research Facility, Melbourne, Australia, using one-fifth of a HiSeq 2000 lane for each library. Following quality filtering (with a low-quality threshold of 0.05, the maximum number of ambiguities set at 2, removal of 2 terminal nucleotides, and a minimum length of 50 bases) of paired-end Illumina reads, the genome sequences were assembled from a minimum of 5.2 × 10^7^ reads using the CLC Genomics Workbench version 5.1 with the scaffolding option selected (minimum contig size, 200 bp). Coverage of the genomes ranged from 836- to 1,208-fold. Total genome size ranged from 5.5 to 6 Mbp. Details of the genome assemblies are presented in Table 1. Scaffolds were split into component contigs at any site of sequence ambiguity (i.e., even a single N) using custom Perl scripts to allow gene annotation using the NCBI Prokaryote Genomes Automatic Annotation Pipeline (PGAAP). Contig relationships were maintained in the GenBank submission by the inclusion of A Golden Path (AGP) file. The output of the PGAAP was manually curated to correct the nomenclature of the type III secretion system effectors in the *P. syringae* genomes.

**TABLE 1:** Genome assembly statistics and accession numbers

Isolate	Assembly size (Mbp)	Scaffold no. (contigs)	*N*_50_^*a*^	*L*_50_ (kbp)*^a^*	Accession no.
*P. syringae* BRIP 34876	6.02	99 (148)	7	232	AMXK00000000
*P. fluorescens* BRIP 34879	5.53	110 (261)	13	150	AMZW00000000
*P. syringae* BRIP 34881	6.12	96 (157)	6	300	AMXL00000000
*P. syringae* BRIP 39023	5.94	35 (71)	6	356	AMZX00000000

a *N*_50_ and *L*_50_ statistics refer to scaffold sequences.

### Nucleotide sequence accession numbers.

These Whole-Genome Shotgun projects have been deposited at DDBJ/EMBL/GenBank under the following accession no.: AMXK00000000 for *P. syringae* isolate BRIP 34876, AMXL00000000 for *P. syringae* isolate BRIP 34881, AMZW00000000 for *P. fluorescens* isolate BRIP 34879, and AMZX00000000 for *P. syringae* isolate BRIP 39023.

## References

[1] Klosterman SJ Subbarao KV Kang S Veronese P Gold SE Thomma BPHJ Chen Z Henrissat B Lee Y-H Park J Garcia-Pedrajas MD Barbara DJ Anchieta A de Jonge R Santhanam P Maruthachalam K Atallah Z Amyotte SG Paz Z Inderbitzin P Hayes RJ Heiman DI Young S Zeng Q Engels R Galagan J Cuomo CA Dobinson KF Ma L-J 2011 Comparative genomics yields insights into niche adaptation of plant vascular wilt pathogens. PLoS Pathog. 7:e1002137 21829347 10.1371/journal.ppat.1002137 PMC3145793

[2] Gardiner DM McDonald MC Covarelli L Solomon PS Rusu AG Marshall M Kazan K Chakraborty S McDonald BA Manners JM 2012 Comparative pathogenomics reveals horizontally acquired novel virulence genes in fungi infecting cereal hosts. PLoS Pathog. 8:e1002952 23028337 10.1371/journal.ppat.1002952 PMC3460631

[3] de Jonge R van Esse HP Maruthachalam K Bolton MD Santhanam P Saber MK Zhang Z Usami T Lievens B Subbarao KV Thomma BPHJ 2012 Tomato immune receptor Ve1 recognizes effector of multiple fungal pathogens uncovered by genome and RNA sequencing. Proc. Natl. Acad. Sci. U. S. A. 109:5110–5115 22416119 10.1073/pnas.1119623109 PMC3323992

